# Optimization of a New Composite Multicellular Plate Structure in Order to Reduce Weight

**DOI:** 10.3390/polym14153121

**Published:** 2022-07-31

**Authors:** György Kovács

**Affiliations:** Faculty of Mechanical Engineering and Informatics, Institute of Manufacturing Science, University of Miskolc, H-3515 Miskolc, Hungary; altkovac@uni-miskolc.hu

**Keywords:** new multicellular plate structure, *FRP* materials, structural optimization, weight saving

## Abstract

Currently, the most important structural design aims are weight reduction, corrosion resistance, high stiffness and vibration damping in several industrial applications, which can be provided by the application of advanced fiber-reinforced plastic (*FRP*) composites. The main research aim was to develop novel and innovative multicellular plate structures that utilize the benefits of lightweight advanced *FRP* and aluminum materials, as well as to combine the advantageous characteristics of cellular plates and sandwich structures. Two new multicellular plate structures were developed for the structural element of a transport vehicle. The first structure consists of carbon-fiber-reinforced plastic (*CFRP*) face sheets and pultruded glass-fiber-reinforced plastic (*GFRP*) stiffeners. The second structure consists of carbon-fiber-reinforced plastic face sheets and aluminum (*Al*) stiffeners. The second main goal of this research was the development of an optimization method of minimal weight for the newly developed all-*FRP* structure and the *CFRP-Al* structure, considering seven design constraints. The third main purpose was to confirm in a real case study that lightweight multicellular composite constructions, optimized by the flexible tolerance optimization method, provide significant weight saving (86%) compared to the all-steel structure. The added value of the research is that optimization methods were developed for the constructed new composite structures, which can be applied in applications where weight saving is the primary aim.

## 1. Introduction

Currently, the biggest challenge for companies is maintaining or increasing their competitiveness in the growing market competition. Furthermore, enterprises have to flexibly adapt to rapidly changing customer demands. In this market, enterprises, on the one hand, have to apply new and modern production technologies, innovative constructions and advanced materials; on the other hand, they have to reduce costs and increase profits. Research and development activity and structural optimization are essential for companies to manufacture competitive final products.

There are many benefits of advanced fiber-reinforced-polymer (*FRP*) composites compared with traditional structural materials, i.e., wood, steel or concrete. Consequently, new construction technologies use *FRP* materials as alternative materials instead of the previously mentioned traditional materials. The advantageous properties of *FRP* composites are low weight, excellent strength-to-weight ratio, good vibration damping, resistance to corrosion and chemicals, good bending stiffness, aesthetic appearance, low maintenance requirement, good design versatility, ease of fabrication and installation, ability to be formed into complex shapes, long overall service life, etc. [1,2].

The most advantageous property of *FRP* composites is their low weight, which makes them more attractive compared to traditional metals, e.g., structural steel due to the possibility of significant weight saving. This weight saving results in *FRP* materials being commonly applied in engineering structures where the main optimization goal is mass reduction, e.g., in the civil, automotive, mechanical, marine, biomedical or aerospace industries [3,4,5].

*FRP* composites consist of two different phase materials, which are base materials (matrix materials) and filler materials (fibers) [6,7]. The fibers provide the required strength in one or more directions. The role of the matrix materials is to hold and protect the filler materials from negative impacts. The polymer matrix can be either thermoset or thermoplastic. There are many types of natural fibers and synthetic fibers. Synthetic fibers are preferred in engineering applications because of their higher performance.

There are several types of fibers; the most commonly used are carbon, glass, basalt or aramid [8,9]. There are also many types of matrixes, e.g., resins, metals, ceramics, etc. [10,11]. The variation of these material constituents is almost extremely high, which provides the required combination of constituents for given applications [12,13].

The most widely applied form of *FRP* composites is laminated *FRP* composite plates. Laminated plates consist of more laminas (layers). Instead of traditional metal structures, these composite laminates are often used because of their high strength and light weight [14,15].

Traditional engineering materials, e.g., steel, are homogenous with the same material properties in all directions, while *FRP* material has different properties in different directions. This means that fiber composites are anisotropic in nature, and their behavior mainly depends on the material properties of their matrix and fiber constituents.

There are many publications that introduce in detail the theories and design principles of the micro- and macromechanics of laminated structures. Based on the literature, it can be summarized that the design procedures for laminated composite structures are more complicated than in the case of homogenous materials [16,17,18]. This complexity means that the design procedure of an *FRP* composite structure should include not only the geometry or shape design of the structure but also the design of the type and ratio of material constituents and fiber orientations.

Several publications discuss the optimization procedures for *FRP* composite constructions, including objective functions and design constraints, as well as the applied optimization algorithms [19,20,21,22]. The most commonly used objective functions are cost, mass, middle deflection, number and order of layers or fiber orientations in the laminate, etc. [23,24,25]. Finite element modeling and experimental measurements are also often discussed in several publications in the literature for the validation of analytical calculations [26,27,28].

The main purpose of this study was to build new multicellular structures that utilize the benefits of advanced lightweight *FRP* and aluminum, as well as to combine the advantageous characteristics of cellular plates and sandwich structures.

Two new multicellular plate structures were constructed. The first structure consists of carbon-fiber-reinforced plastic (*CFRP*) face sheets and pultruded glass-fiber-reinforced plastic (*GFRP*) stiffeners. The second construction consists of carbon-fiber-reinforced plastic face sheets and aluminum (*Al*) stiffeners. (These investigated new structures are depicted in Section 2.) The constructed multicellular plate structures are the combination of different lightweight materials (i.e., *CFRP*, *GFRP*, *Al*), as well as the combination of different structures (i.e., traditional cellular plates and traditional sandwich structures). Consequently, the combination of the benefits of various lightweight materials and structural components can be more advantageous than using constituent materials and structural elements individually.

*CFRP* and *GFRP* composite materials were applied; furthermore, aluminum has low weight, high chemical and corrosion resistance, high strength and high damping performance. Therefore, these attractive properties can fulfill the special requirements of unique engineering applications.

The developed multicellular plate is a “sandwich-like” construction, in which the face sheets are manufactured from advanced *CFRP* materials (like the face sheets of traditional sandwich structures), but the inner core (stiffeners) between the two face sheets comprises tubes (like the stiffeners of traditional cellular plate structures).

On the one hand, traditional *FRP* sandwich structures consist of two *FRP* face sheets and a low-density inner core. The most commonly applied cores are honeycomb cells or foams. The most advantageous properties of *FRP* sandwich constructions are high stiffness, high strength-to-weight ratio, perfect design versatility, excellent vibration damping, fast and easy manufacturing, etc. [29,30,31]. Several publications are available in the field of the design, structural optimization and practical usage of sandwich structures [32,33].

On the other hand, traditional cellular plate structures are built from one or two metal deck plates and welded metal tubes. Cellular plate structures, because of the two metal deck plates and tubes, provide high strength and stiffness, as well as relatively low cost. The optimization processes for cellular plate structures are more complicated due to the complex structural geometry as compared to the optimization processes for monolithic structures [34,35,36].

- Stages of the Research and the Main Parts of the Publication

The primary goal of this study was to construct new multicellular plate structures that utilize the benefits of lightweight advanced *FRP* and aluminum, as well as to combine the advantageous characteristics of cellular plates and sandwich structures.

Two new multicellular plate structures were developed. The first construction was built from *CFRP* face sheets and pultruded *GFRP* tubes. The second construction was built from *CFRP* face sheets and *Al* tubes. An all-steel multicellular structure is also presented to show that the application of a lightweight composite structure, instead of the steel construction, results in significant weight saving.

The second main aim was to develop and introduce structural optimization procedures for the two newly developed multicellular plate structures. A detailed weight objective function was developed for both new structures; furthermore, seven design constraints were developed and taken into consideration during the optimization.

In this article, the application of the developed optimization methods in a real example is also introduced, which is a structural component of a road truck trailer. The optimal construction of this structural element is defined in the case of all-*CFRP-GFRP* and *CFRP-Al* structures by the flexible tolerance optimization (FTO) method. It is confirmed that constructed optimal multicellular plate structures provide many advantages compared to the all-steel multicellular plate structure. In the real case studies, significant weight saving can be achieved by the application of advanced *FRP* composite and *Al* materials due to their low density.

The novelty and main contribution of this study are that a weight minimization method considering seven design constraints was developed for the two newly developed multicellular plate structures: (1) *CFRP* face sheets with pultruded *GFRP* stiffeners, and (2) *CFRP* face sheets with *Al* stiffeners. The effectiveness of the developed optimization method was approved by the structural optimization of the composite structural element of a road truck trailer, which resulted in 86% weight saving compared to the all-steel structural element. This significant weight saving results in lower fuel consumption of the vehicle. Thus, lower fuel consumption causes less environmental damage, providing sustainable transportation.

## 2. Materials and Methods: Construction of New Composite Multicellular Plate Structures

The main aim of this research is the structural optimization of the two newly developed multicellular plate structures, providing lightweight constructions for a given structural component of a road truck trailer. In addition to weight saving, corrosion resistance is also an important advantageous characteristic of the developed structures.

The developed new multicellular plate structure is novel because the structural model combines different materials and different structural elements. The multicellular plate structure combines the general properties of sandwich structures and cellular plate structures.

**Figure 1:**
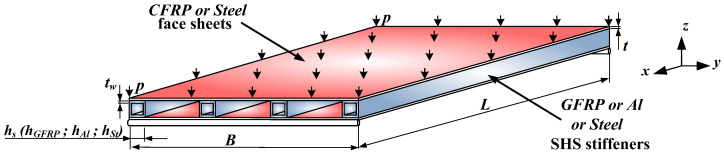
Investigated multicellular plate structure.

In this study, three different multicellular plate structures are analyzed and optimized. The investigated structures consist of upper and lower face sheets and stiffeners between them, as shown in Figure 1. The geometry and loading conditions are the same in the case of the three investigated different multicellular plate structures, but the materials and geometries of the structural components are different.

The investigated multicellular plate structure is simply supported. The length and width of the structure are *L* = 2250 mm and *B* = 2000 mm, respectively. *t* is the thickness of the face sheets, *h* is the height and width of the stiffeners and *t_W_* is the wall thickness of the stiffeners. In the calculations, depending on the material of the stiffeners, “*h*” is used as *h_GFRP_* in the case of the *GFRP* stiffeners, *h_Al_* in the case of the *Al* stiffeners and *h_St_* in the case of the steel stiffeners. Uniformly distributed loading (*p* = 3.5⋅10^−3^ MPa) acts on the upper face sheet of the multicellular plate structure.

The following three constructions are investigated and optimized for a given element of a road truck trailer:

1. Laminated carbon-fiber-reinforced plastic (*CFRP*) face sheets with glass-fiber-reinforced plastic (*GFRP*) square hollow section (SHS) stiffeners;
2. Laminated carbon-fiber-reinforced plastic (*CFRP*) sheets with aluminum (*Al*) SHS stiffeners;
3. Steel face sheets with steel SHS stiffeners.

During the structural optimization, the optimal number of the stiffeners, the optimal geometry of the stiffeners and the thickness (number of layers in the laminate) of the face sheets have to be defined for the three different constructions.

### 2.1. The First Multicellular Plate Structure Consists of Laminated Carbon-Fiber-Reinforced Plastic (CFRP) Face Sheets and Glass-Fiber-Reinforced Plastic (GFRP) SHS Stiffeners

**Figure 2:**
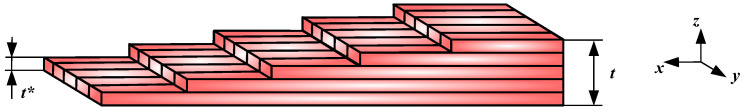
Laminate of the face sheet.

The first investigated multicellular composite construction is novel since all the materials of the structural elements are lightweight fiber-reinforced plastic composites. The structure is built from two laminated *CFRP* face sheets and pultruded *GFRP* SHS stiffeners. Laminated composite face sheets are constructed of thin layers (lamina) stacked together (Figure 2). The face sheets and stiffeners are connected with adhesive bonding.

*CFRP* material was selected for the face sheets because it is one of the most commonly used lightweight materials in engineering applications. This material provides high weight saving and a high strength-to-weight ratio.

Pultruded *GFRP* square hollow section stiffeners are additional structural components that also provide high weight saving, high stiffness and relatively lower cost of glass fibers compared to carbon fibers.

- Properties of the CFRP Face Sheets

The face sheet is a laminated plate structure (Figure 2). The layers are the product of the Hexcel Composites Company. All of the material properties are provided by the manufacturer [37]. The volume fraction of the carbon fiber is 61%, while the volume fraction of the epoxy matrix is 39% in a lamina. All of the fibers are placed in the *x*-direction. The thickness of a lamina is t* = 0.2 mm. The material properties are the following: Young’s modulus in the *x*-direction is *E_x_* = *E_C_* = 120 GPa, while in the *y*-direction, it is *E_y_* = 9 GPa; Poisson’s ratios are *ν_xy_* = 0.25 and *ν_yx_* = 0.019; and density is *ρ_C_* = 180 g/m^2^. The thickness of the laminated plate (*t*) is the sum of the thicknesses of the layers (t*). The total thickness of the laminate can be calculated as *t* = n·t*, where *n* is the number of layers in the laminate.

- Properties of the Pultruded GFRP SHS Stiffeners

Properties of the pultruded *GFRP* stiffener: Young’s modulus is *E_G_* = 40 GPa, and the density is *ρ_G_* = 2⋅10^6^ g/m^3^.

Table 1 shows the sizes (*h_GFRP_*, *t_W_*) of the available pultruded *GFRP* profiles that were taken into consideration during the structural optimization.

### 2.2. The Second Multicellular Plate Structure Consists of Laminated Carbon-Fiber-Reinforced Plastic (CFRP) Face Sheets and Aluminum (Al) SHS Stiffeners

The second investigated multicellular composite structure is also a lightweight structure because it is constructed from two laminated *CFRP* sheets and aluminum SHS tubes. The two face sheets and stiffeners are also connected with adhesive bonding.

- Properties of the CFRP Sheets

The face sheet is also a laminated plate structure. The applied layers are the same as in the case of the face sheets of the previously mentioned (in Section 2.1) first multicellular plate structure.

- Properties of the Al SHS Stiffeners

*Al* SHS tubes, like the other structural components, also provide high weight saving, relatively high stiffness and low cost.

Properties of the *Al* stiffener: Young’s modulus is *E_Al_* = 70 Gpa, and the density is *ρ_Al_ =* 2.7·10^−6^ kg/mm^3^.

Table 2 shows the sizes of the stiffeners (*h_Al_, t_w_*) that were taken into consideration during the structural optimization.

### 2.3. The Third Multicellular Plate Structure Constructed from Steel Deck Plates and Steel SHS Stiffeners

The third investigated multicellular structure is a traditional all-steel structure. The investigated construction consists of two steel face sheets and steel tubes welded between the upper and lower deck plates using arc-spot welding technology. This structure was earlier investigated and optimized by Jármai et al. [38].

The aim of the introduction of this all-steel structure is to show that the application of advanced lightweight composite materials and structural elements results in significant weight saving. Therefore, the two previously mentioned (Section 2.1 and Section 2.2) composite multicellular structures are compared with this all-steel structure to emphasize the advantages of modern composite materials.

## 3. Development of the Optimization Methods for the New Multicellular Plate Structures

The main aim of this research was to develop optimization methods for the first and second newly developed composite multicellular plate structures, which can utilize the advantageous characteristics of *FRP* materials.

During the development of the optimization methods, the weight objective function, as well as the design constraints, has to be defined. The design variables to be optimized are the following: number of layers of the laminated *CFRP* face sheets (*n*), number of the *GFRP* or *Al* SHS stiffeners (*n_s_*), and height (*h_GFRP_/h_Al_*) and wall thickness (*t_W_*) of the *GFRP* or *Al* SHS stiffeners. The optimization task was solved by the FTO optimization method.

### 3.1. Optimization Method Developed for the First Multicellular Plate Structure Constructed from Laminated CFRP Sheets and GFRP SHS Stiffeners and for the Second Multicellular Plate Structure Constructed from Laminated CFRP Sheets and Al SHS Stiffeners

#### 3.1.1. Weight Objective Function

The main design aim in the case of the structural element of the road truck trailer is that the structure has to be lightweight. Therefore, the purpose of the research was the development of a detailed weight objective function for the newly developed multicellular composite plate structures.

The weight of the newly developed multicellular plate structures (*m*_1,2_) is the sum of the weight of the structural components, i.e., the two face sheets and stiffeners. The weight of the adhesive bonding can be neglected because one tube (380 mL) is enough for the bonding of 4 m^2^. In our case, the surface of the structural elements to be bonded is approximately 1.8 m^2^. The adhesive bonding (HexBond 21ST1007) is the product of the Hexcel Composites Company (Salt Lake, UT, USA). The shear strength of the adhesive is 18.6 Mpa, while the compression strength is 36.8 Mpa [37]. The calculation of the total weight is the following:

(1) $$m_{1,2}=2\rho_{C}\left[BL(nt^{*})\right]+n_{S}\rho_{S}\left[L(4h_{S}t_{W}-4t_{W}^{2})\right]$$

where *B* is the width of the structure, *L* is the length of the structure, *n* is the number of layers in the laminated face sheets, t* is the thickness of layers, $\rho_{C}$ is the density of the *CFRP* laminate, *n_s_* is the number of the stiffeners, $\rho_{s}$ is the density of the stiffeners, *h_S_* is the height and width, and *t_W_* is the wall thickness of the SHS stiffeners.

In the case of the first structure, where pultruded *GFRP* SHS stiffeners are used, $\rho_{s}$ has to be replaced by $\rho_{G}$, which is the density of the pultruded *GFRP* stiffener; furthermore, *h_S_* has to be replaced by *h_GFRP_*, which is the height of the *GFRP* SHS stiffener.

In the case of the second structure, where aluminum SHS stiffeners are applied, $\rho_{s}$ has to be replaced by $\rho_{Al}$, which is the density of the aluminum stiffener; furthermore, *h_S_* has to be replaced by *h_Al_*, which is the height of the *Al* SHS stiffener.

#### 3.1.2. Design Constraints

The calculation methods for the considered seven design constraints are detailed in this subsection.

1. *Deflection of the Multicellular Plate Structure*

Based on the previous test measurements [39], it can be concluded that there is a relative movement between the face sheet and stiffener under bending. This relative movement of the structural elements resulted in an additional deflection, which can be defined as the second part of Equation (2). The value of this relative movement depends on the differences in predicted stress in the middle line of the flange of the pultruded stiffener and the stress at the same point of the face sheet. This difference in stress (Δσ) causes differences in the equivalent applied moment (Δ*M*).

(2) $$w_{\max}=\frac{5p\;L^{4}}{384(E_{C}I_{C}+E_{S}n_{S}I_{S})}+\frac{5\Delta M\;L^{2}}{48(E_{C}I_{C}+E_{S}n_{S}I_{S})}\leq \frac{L}{200}$$

where *w*_max_ is the occurring maximal middle deflection of the structure; *I_C_* and *I_S_* are the moments of inertia for the *CFRP* face sheet and stiffener; *E_C_* and *E_S_* are the moduli of elasticity for the *CFRP* face sheet and stiffener, respectively; and *n_S_* is the number of stiffeners.

In the case of the first structure, *I_S_* has to be replaced by *I_G_*, which is the moment of inertia for the *GFRP* stiffener; furthermore, *E_S_* has to be replaced by *E_G_*, which is the modulus of elasticity of the *GFRP* stiffener.

In the case of the second structure, *I_S_* has to be replaced by *I_Al_*, which is the moment of inertia for the aluminum stiffener; furthermore, *E_S_* has to be replaced by *E_Al_*, which is the modulus of elasticity of the *Al* stiffener.

2. *Stress Occurring in the Laminated CFRP Sheet*

The moment occurring in the multicellular plate construction is divided between the stiffeners and face sheet. The moment acting on the face sheet (*X_C_M*) and the moment acting on the stiffeners (*X_S_M*) can be calculated. The calculation of the stress in the laminated face sheet is the following:

(3) $$\frac{X_{C}M}{I_{C}}\cdot \frac{h_{S}+nt^{*}}{2}\leq \sigma_{C.all}$$

where *X_C_M* is the ratio of the moment occurring in the face sheet, $M=\frac{pL^{2}}{8}$, $X_{C}=\frac{E_{C}I_{C}}{E_{S}n_{S}I_{S}+E_{C}I_{C}}$, $\sigma_{C.all}=\frac{\sigma_{T}}{\gamma_{C}}$ is the allowable stress, σ*_T_* is the tensile strength of the face sheet, and γ*_C_*= 2 is the factor of safety.

In the case of the first structure, *h_S_* has to be replaced by *h_GFRP_*; furthermore, *E_S_* and *I_S_* have to be replaced by *E_G_* and *I_G_*.

In the case of the second structure, *h_S_* has to be replaced by *h_Al_*; furthermore, *E_S_* and *I_S_* have to be replaced by *E_Al_* and *I_Al_*.

3. *Stress Occurring in the Stiffener*

The maximal stress occurring in the *GFRP* stiffener has to be lower than the allowable stress:

(4) $$\frac{X_{S}M}{n_{S}I_{S}}\cdot \frac{h_{S}}{2}\leq \sigma_{S.all}$$

where $X_{S}=\frac{E_{S}n_{S}I_{S}}{E_{S}n_{S}I_{S}+E_{C}I_{C}}$, *X_S_M* is the ratio of the moment acting on the stiffener, and $\sigma_{S.all}$ is the allowable stress for the stiffener.

In the case of the first structure, *h_S_*, *E_S_, I_S_* and $\sigma_{S.all}$ have to be replaced by *h_GFRP_*, *E_G_, I_G_* and $\sigma_{G.all}$. In the case of the second structure, *h_S_*, *E_S_, I_S_* and $\sigma_{S.all}$ have to be replaced by *h_Al_*, *E_Al_, I_Al_* and $\sigma_{Al.all}$. $\sigma_{Alall}=\frac{f_{y}}{\gamma_{Al}}$ is the allowable stress in the case of the *Al*, *f_y_* is the yield stress of the aluminum, and γ*_Al_* = 2 is the factor of safety.

4. *Buckling of the CFRP Face Sheet between the Stiffeners*

The buckling of the face sheets between the stiffeners is a common failure mode of thin plates. Therefore, this buckling has to be limited according to [40]:

(5) $$\left(\frac{b_{c}}{nt*}\right)\leq \sqrt{\frac{\pi^{2}}{6\sigma_{\max}\left(1-\nu_{xy}\nu_{yx}\right)}\left[\sqrt{E_{x}E_{y}}+E_{x}\nu_{xy}+2G_{xy}\left(1-\nu_{xy}\nu_{yx}\right)\right]}$$

where *b_c_* is the width of the face sheet between the two SHS stiffeners; *E_x_, E_y_, G_xy_,* ν*_xy_* and ν*_yx_* are the material properties of the laminate; and σ*_max_* is the maximal stress in the laminate.

5. *Buckling of the Webs of Stiffeners*

The buckling of the webs of the stiffeners also has to be considered during the optimization. Therefore, this buckling has to also be limited:

(6) $$\frac{h_{S}}{t_{w}}\leq 42\sqrt{\frac{235E_{S}}{240E_{St}}}$$

where *E_S_* and *E_St_* are Young’s moduli of the stiffeners and steel.

In the case of the first structure, *h_S_* and *E_S_* have to be replaced by *h_GFRP_* and *E_G_*, while in the case of the second structure, *h_S_* and *E_S_* have to be replaced by *h_Al_* and *E_Al_.*

6. *Eigenfrequency of the Multicellular Plate Structure*

The eigenfrequency design constraint also has to be taken into consideration during the optimization. This constraint can be formalized as the following:

(7) $$f_{1}=\frac{\pi }{2L^{2}}\sqrt{\frac{10^{3}(E_{S}I_{S}+E_{C}I_{C})}{m}}\geq f_{0}$$

where *m* is the unit mass per meter for the multicellular plate construction (kg/m), and *f_0_* is the allowable eigenfrequency (in our case, 50 Hz).

In the case of the first structure, *E_S_* and *I_S_* have to be replaced by *E_G_* and *I_G_* and in the case of the second structure by *E_Al_* and *I_Al_.*

7. *Limitations for the Design Variables to be Optimized*

The four limitations mentioned below were defined for the design variables considering manufacturing and economical aspects.

In the case of the first structure, where pultruded *GFRP* SHS stiffeners are used, the following limitations have to be taken into consideration:

16 ≤ *n* ≤ 32 (pcs)

4 ≤ *n_s_* ≤ 20 (pcs) (8)

25 ≤ *h_GFRP_* ≤ 100 (mm)

2.5 ≤ *t_W_* ≤ 10 (mm)

In the case of the second structure, where aluminum SHS stiffeners are applied, the following limitations have to be considered:

16 ≤ *n* ≤ 32 (pcs)

7 ≤ *n_s_* ≤ 20 (pcs) (9)

10 ≤ *h_Al_* ≤ 100 (mm)

2 ≤ *t_W_* ≤ 6 (mm)

### 3.2. Optimization Method for the Third Multicellular Plate Structure Consists of Steel Face Sheets and Steel SHS Stiffeners

This third investigated multicellular structure, introduced in Section 2.3, is a traditional all-steel (*f_y_* = 355 MPa) structure consisting of steel stiffeners welded between the upper and lower face sheets using arc-spot welding technology. This structure was optimized by Jármai et al. [38]. The width and length (*B*, *L*) of the construction to be optimized, as well as the loading condition (*p*), were equal to the two above-mentioned composite multicellular plate structures introduced in Section 2.1 and Section 2.2.

#### 3.2.1. Weight Objective Function

The weight objective function for the all-steel multicellular plate structure was introduced in [38].

The weight of the steel multicellular plate structure (*m*_3_) included the weight of the steel deck plates and steel stiffeners. The calculation of the total weight was the following:

(10) $$m_{3}=2\rho_{S}\left(BLt\right)+n_{s}\rho_{S}\left[L(4h_{St}t_{w}-4t_{w}^{2})\right]+m_{add}$$

where $\rho_{S}$ is the density of the steel, *t* is the thickness of the steel deck plate, *h_st_* and *t_W_* are the sizes of the steel stiffeners, and *m_add_* is the additive mass of the welding.

#### 3.2.2. Design Constraints

Three design constraints were considered during the optimization procedure: (1) constraint for eigenfrequency of the total construction, (2) constraint for stability due to compression and bending, and (3) stress constraint for the upper face sheet.

The optimization problem was solved by Rosenbrock’s hill-climbing mathematical programming method [38].

## 4. Results of the Structural Optimizations

In this section, the minimal weight optimization results are introduced for the three investigated multicellular plate structures.

The optimization of the newly constructed multicellular composite plate structures was carried out by the FTO method for minimal weight.

The FTO method is a constrained random search technique. The method applies two searches (an external search and an internal search) to satisfy feasibility constraints [41].

Minimize: *f(x) x* ∈ R^n^

While: *Φ*_(*k*)_ – *T*_(*x*)_ ≥ 0 (11)

where $\Phi_{\left(k\right)}$ is the criteria for flexible tolerance for the viability at stage *k* of the search, and *T(x)* is a positive function for the equality and inequality design constraints [42].

The main purpose of the design was the construction of a given application that provides minimal weight. The result of the weight optimization of the two newly developed composite multicellular plate constructions investigated, applying the weight objective function (Equation (1)) and the seven above-mentioned design constraints (Equations (2)–(9)), is summarized in Table 3 and Table 4. The result of the weight optimization of the traditional all-steel multicellular plate construction, applying the weight objective function (Equation (10)) and the three design constraints mentioned in Section 3.2.2, is summarized in Table 5.

Every line of the Table 3, Table 4, Table 5 and Table 6. shows a local optimum in the case of a given number of *CFRP* face sheets, i.e., the optimal numbers and sizes of the stiffeners, as well as the total weight of the different optimal structures. The global optimum, of which the structure provides minimal weight, is highlighted by bold numbers and red color.

1. *Results of the Weight Optimization for the First Multicellular Plate Structure Constructed from Laminated CFRP Sheets and GFRP SHS Stiffeners*

**Figure 3:**
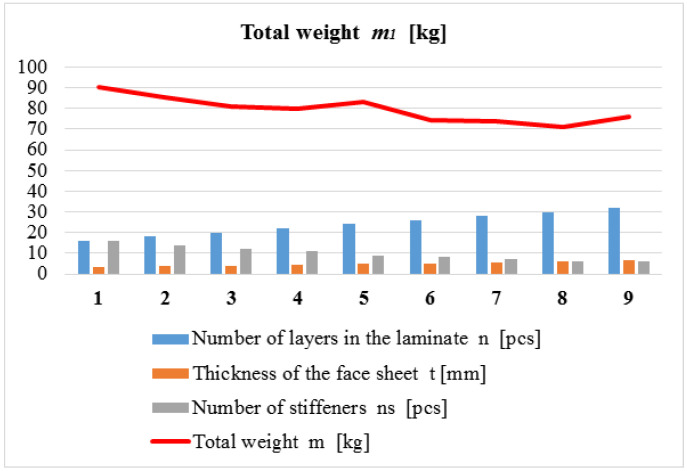
Result of the weight optimization.

Table 3 and Figure 3 show the optimal construction that provides minimal weight in the case of the multicellular plate structure consisting of laminated *CFRP* face sheets and *GFRP* SHS stiffeners.

**Table 3:** Result of the weight optimization.

Number of Layers in the Laminate, *n* (Pieces)	Thickness of Face Sheets, *t* (mm)	Optimal Sizes and Numbers of Stiffeners			Weight, *m*_1_ (kg)
		*h_GFRP_* (mm)	*t_W_* (mm)	*n_s_* (Pieces)	
16	3.2	60	4	16	90.43
18	3.6	60	4	14	85.61
20	4.0	60	4	12	80.78
22	4.4	60	4	11	79.99
24	4.8	60	4	9	83.23
26	5.2	60	4	8	74.38
28	5.6	60	4	7	73.58
**30**	**6.0**	**60**	**4**	**6**	**72.79**
32	6.4	60	4	6	76.03

If the number of layers of the face sheet increases, the total weight of the structure decreases; thus, it results in weight saving. Based on the weight optimization, it can be summarized that the optimal all-*FRP* multicellular plate structure, which provides minimal weight (72.79 kg), has 30-layered laminated *CFRP* face sheets and 6 pieces of 60 × 60 × 4 mm pultruded *GFRP* stiffeners.

2. *Results of the Weight Optimization for the Second Multicellular Plate Constructed from Laminated CFRP Composite Face Sheets and Al SHS Stiffeners*

**Figure 4:**
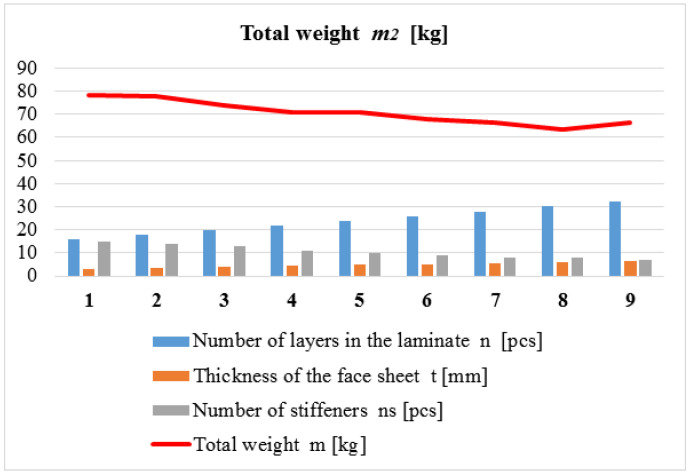
Result of the weight optimization.

Table 4 and Figure 4 show the optimal construction that provides minimal weight in the case of the multicellular plate structure consisting of laminated *CFRP* face sheets and *Al* SHS stiffeners.

**Table 4:** Result of the weight optimization.

Number of Layers in the Laminate, *n* (Pieces)	Thickness of Face Sheets, *t* (mm)	Optimal Sizes and Numbers of Stiffeners			Weight, *m*_2_ (kg)
		*h_Al_* (mm)	*t_W_* (mm)	*n_s_* (Pieces)	
16	3.2	60	2.5	15	78.317
18	3.6	60	2.5	14	78.064
20	4.0	55	2.5	13	73.862
22	4.4	55	2.5	11	70.723
24	4.8	55	2.5	10	70.8
26	5.2	50	2.5	9	68.1
28	5.6	50	2.5	8	66.445
**30**	**6.0**	**45**	**2**	**8**	**65.32**
32	6.4	45	2	7	66.469

If the number of layers of the face sheet increases, the total weight of the structure decreases; thus, it results in weight saving. Based on the weight optimization, it can be summarized that the optimal multicellular plate constructed from laminated *CFRP* composite sheets and *Al* SHS stiffeners, which provide minimal weight (65.32 kg), has 30-layered laminated *CFRP* face sheets and 8 pieces of 45 × 45 × 2 mm *Al* stiffeners.

3. *Results of the Weight Optimization for the Third Multicellular Plate Structure Consists of Steel Face Sheets and Steel SHS Stiffeners*

Table 5 shows the optimal steel construction, i.e., the number and sizes of the optimal steel stiffeners, as well as the optimal thickness of the steel face sheets and the total weight of the optimal structure [38].

**Table 5:** Result of the weight optimization.

Thickness of Face Sheets, *t* (mm)	Optimal Sizes and Number of Stiffeners			Weight, *m*_3_ (kg)
	*h_St_* (mm)	*t_W_* (mm)	*n_s_* (Pieces)	
5	30	2	4	1105
2.5	50	2	5	629
**2**	**40**	**2**	**6**	**517**
2	40	2	7	533
2	40	2	8	548

It can be seen that the weight of the steel structure is extremely high compared to the previous two composite constructions. The optimal steel multicellular plate structure, which provides minimal weight (517 kg), has 6 pieces of 40 × 40 × 2 mm steel stiffeners and face sheets with 2 mm thicknesses.

## 5. Comparison of the Optimization Results for the Three Weight-Optimized Multicellular Plate Structures

The aim of the research was the structural optimization of newly developed multicellular plate structures providing lightweight constructions for a given structural component of a road truck trailer. Therefore, optimization methods were developed for the first and second newly developed composite multicellular plate structures, which can utilize the advantageous characteristics of the *FRP* composite materials.

Data for the three weight-optimized multicellular plate structures are compared in Table 6. The constructed multicellular plate structures consisting of *CFRP* face sheets with *GFRP* or *Al* stiffeners provide many advantages compared to the all-steel multicellular plate structure. The most important advantage is that significant weight saving can be realized by the usage of advanced *FRP* materials, which have low density.

**Table 6:** Comparison of the three weight-optimized multicellular plate structures.

	Thickness of Face Sheets, *t* (mm)	Number of Layers in the Face Sheets,	Optimal Sizes and Number of Stiffeners			Weight, *m* (kg)	Weight Saving (%)
		*n* (Pieces)	*h* (mm)	*t_W_* (mm)	*n_s_* (Pieces)		
**1. Laminated *CFRP* face sheets and pultruded *GFRP* stiffeners**	6	30	60	4	6	72.79	−85.92% (14.08%)
**2. Laminated CFRP face sheets and *Al* stiffeners**	**6**	**30**	**45**	**2**	**8**	**65.32**	**−87.37%** (12.63%)
**3. Steel face sheets and steel SHS stiffeners**	2	-	40	2	6	517	100%

It can be concluded, based on the three compared weight-optimized structures, that significant weight saving can be achieved by the application of *FRP* composites (Table 6). The application of the multicellular plate structure constructed from laminated *CFRP* composite sheets and *GFRP* SHS stiffeners provides **85.92% weight saving**, while the multicellular plate structure constructed from laminated *CFRP* composite sheets and *Al* SHS stiffeners provides **87.37% weight saving** instead of the application of the all-steel multicellular plate structure. This significant weight saving of the component of the road truck trailer body results in a reduction of fuel consumption of the vehicle, as well as the reduction of environmental damage.

## 6. Conclusions

- The main results and conclusions of the study can be summarized in the following points:
  1. Two multicellular plate structures were newly constructed, which utilize the benefits of lightweight advanced *FRP* and aluminum; the advantageous characteristics of cellular plates and sandwich structures were combined.

The first structure was constructed from *CFRP* composite sheets and pultruded *GFRP* stiffeners. The second structure was constructed from *CFRP* composite sheets and aluminum stiffeners. An all-steel multicellular structure was also presented to show that the application of a lightweight composite structure, instead of the steel construction, results in significant weight saving.

  2. New structural optimization methods were developed for the two newly developed multicellular plate structures. The newly developed optimization method for the first all-*FRP* structure was detailed in Section 3.1, while the optimization method for the second *CFRP-Al* structure was introduced in Section 3.2.

A detailed weight objective function was developed for both new structures; furthermore, seven design constraints were developed and considered during the optimization: (1) middle deflection of the multicellular plate construction; (2) stress occurring in the laminated *CFRP* composite sheet; (3) stress occurring in the *GFRP* or in the *Al* stiffener; (4) plate buckling of the *CFRP* sheet between the stiffeners; (5) buckling of the webs of the *GFRP* or the *Al* stiffeners; (6) eigenfrequency of the multicellular plate structure; (7) limitations for the design variables to be optimized. The optimization tasks were carried out by the FTO optimization method.

  3. The developed optimization methods were applied in a real example, which was the optimization of a structural component of a road truck trailer. The optimal construction of this structural element was defined in the case of the all-composite *CFRP-GFRP* and *CFRP-Al* structures by the application of the FTO optimization method (Section 4). It was confirmed that the constructed optimal multicellular plate structures provide many advantages compared to the all-steel multicellular plate structure. In the real case studies, significant weight saving can be achieved by the application of advanced *FRP* composite and *Al* materials due to their low density (Table 6). The multicellular plate structure constructed from laminated *CFRP* face sheets and *GFRP* SHS stiffeners provides 86% weight saving, while the multicellular plate structure constructed from laminated *CFRP* face sheets and *Al* SHS stiffeners provides 87% weight saving instead of the application of the all-steel multicellular plate structure. It can be concluded that gained weight saving is near the same in the case of both optimal lightweight multicellular plate constructions.

- The novelty and main contribution of the study are that a weight minimization method considering seven design constraints was developed for the two newly developed multicellular plate structures: (1) *CFRP* face sheets with pultruded *GFRP* SHS stiffeners; (2) *CFRP* face sheets with aluminum SHS stiffeners. The efficiency of the developed method was confirmed by the structural optimization of the composite structural element of a road truck trailer, which resulted in significant weight saving compared to the all-steel structural element. This significant weight saving results in lower fuel consumption of the vehicle. Thus, the lower fuel consumption causes less environmental damage providing sustainable transportation. The further advantageous characteristic of the developed structures is corrosion resistance, which is also very important in many practical applications.

The newly constructed multicellular plate structures can be applied not only as elements of transport vehicles but also in many other industrial applications, e.g., elements of transport containers or building constructions or elements of bridges.

In future research, more complex multicellular plate structures with other materials and other structural elements can be investigated and optimized, based on the developed optimization methods, for other new industrial applications. Additionally, further constraints and optimization algorithms will be applied during the structural optimization.

## Figures and Tables

**Table 1:** Geometries of the available pultruded *GFRP* SHS stiffeners.

*h_GFRP_* (mm)	25	30	38	40	50	60	75	100
*t_w_* {mm)	2.5	2.5 5	3 4	5 6	3 4 5 6	4 5 8	6 9	6 8 10

**Table 2:** Sizes of the available *Al* SHS stiffeners.

*h_Al_* (mm)	15	20	25	30	34	35	40	45	50	60	70	80	90	100
*t_w_* (mm)	1.5 2	1.5 2	1.5 1.8 2 2.5 3	1.5 2 3	2 2.5 3	2 3	1.5 2 2.5 3 4	2 2.5	1.5 2 2.5 3 3.5 4 5	2 2.5 3 4	4 4.5	2 2.5 3 4 5 6	4	4

## Data Availability

Not applicable.
